# Supplementary figures and images for: Distinct CED-10/Rac1 domains confer context-specific functions in development
Source: PLoS Genet. 2018 Sep 28;14(9):e1007670. doi: 10.1371/journal.pgen.1007670 (PMC6179291; doi:10.1371/journal.pgen.1007670)

A

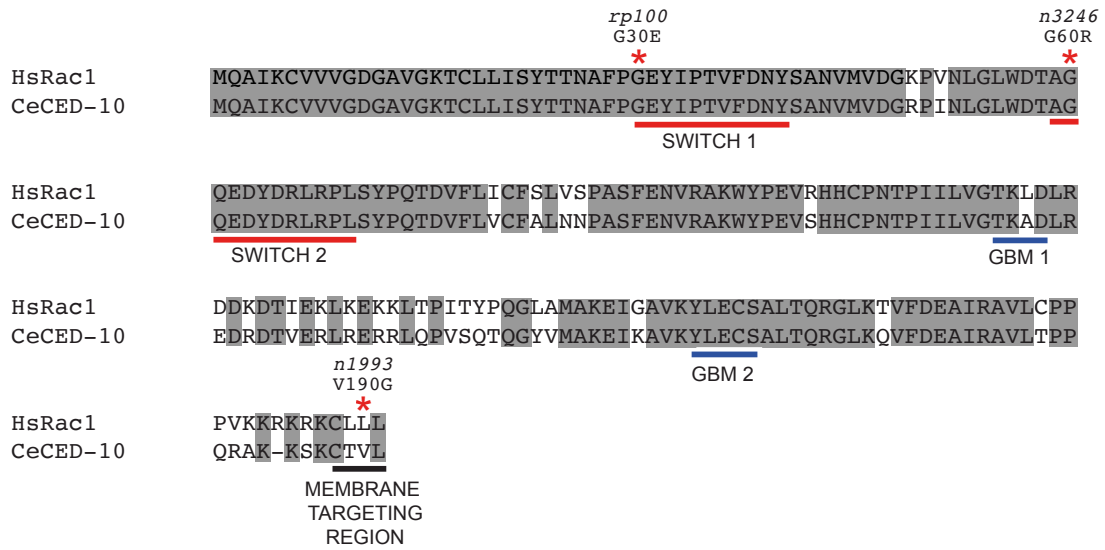

B

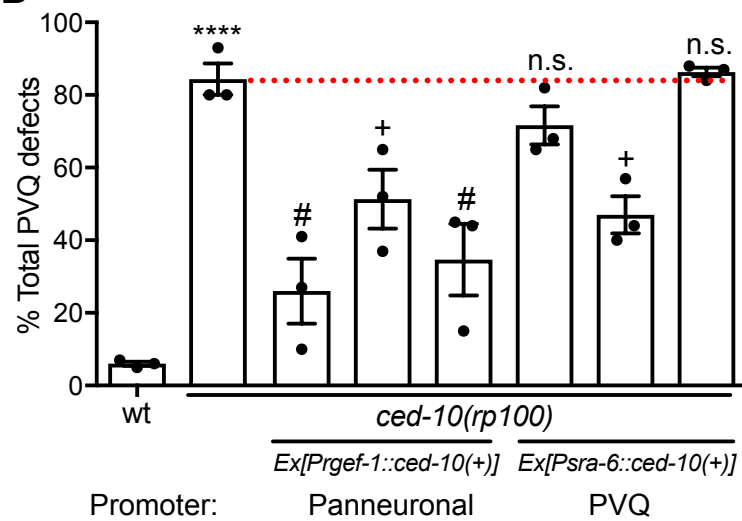

C

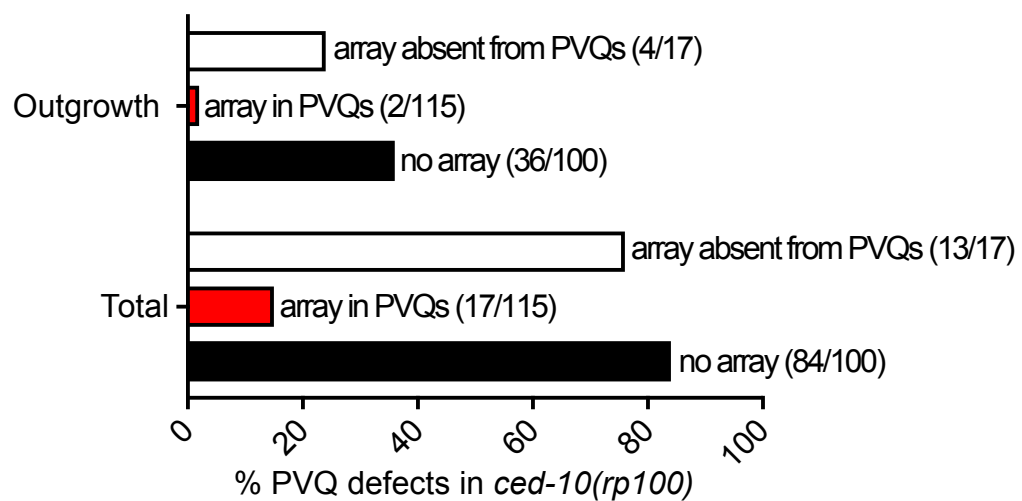

Supplement: S1 Fig — ced-10(rp100) rescue and mosaic analysis. (A) Alignment of the amino acid sequences of Homo sapiens Rac1 (HsRac1) and C. elegans CED-10 (CeCED-10). The ced-10 point mutants used in this study are marked with an asterisk: rp100 (G30E - Switch 1 region), n3246 (G60R - Switch 2 region) and n1993 (V190G - membrane targeting region). (B) Quantification of pan-neuronal and PVQ-specific transgenic rescue of total PVQ defects of ced-10(rp100) animals with ced-10 cDNA (ced-10(+)). Data are expressed as mean ±SD and statistical significance was assessed using one-way ANOVA, with Tukey’s multiple comparison test. ****<0.0001, comparing wild type and rp100 animals; #<0.0001 and +<0.006 and comparing the three transgenic rescue lines to ced-10(rp100). n.s. not statistically significant from rp100 animals. n = 90 per strain, each dot represents independent scoring replicates. (C) An unstable transgenic array containing Prgef-1::ced-10(+), Psra-6::mCherry and Pmyo-2::mCherry plasmids was expressed in ced-10(rp100); oyIs14 animals. PVQ outgrowth and guidance was scored in animals in which the array was absent (Psra-6::mCherry, Pmyo-2::mCherry negative), present in the PVQs (Psra-6::mCherry, Pmyo-2::mCherry positive) or missing from the PVQs (Psra-6::mCherry negative, Pmyo-2::mCherry positive). Number of animals scored is shown in parentheses. (PDF) [file pgen.1007670.s001.pdf]

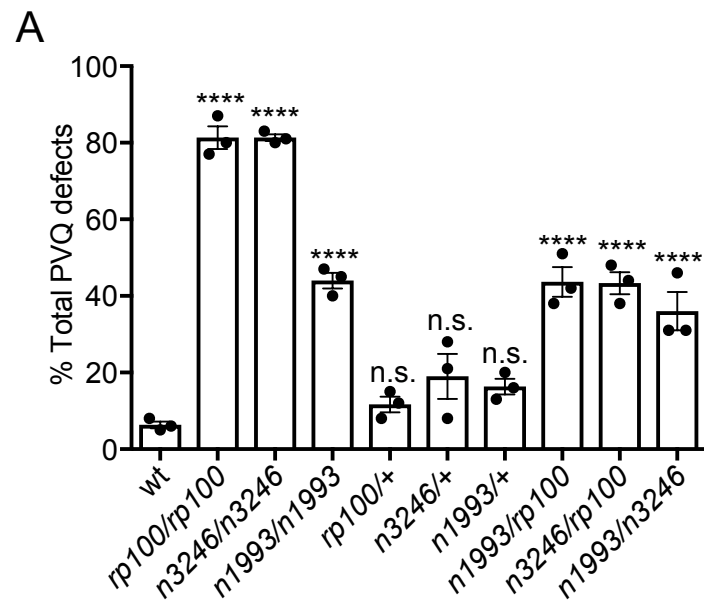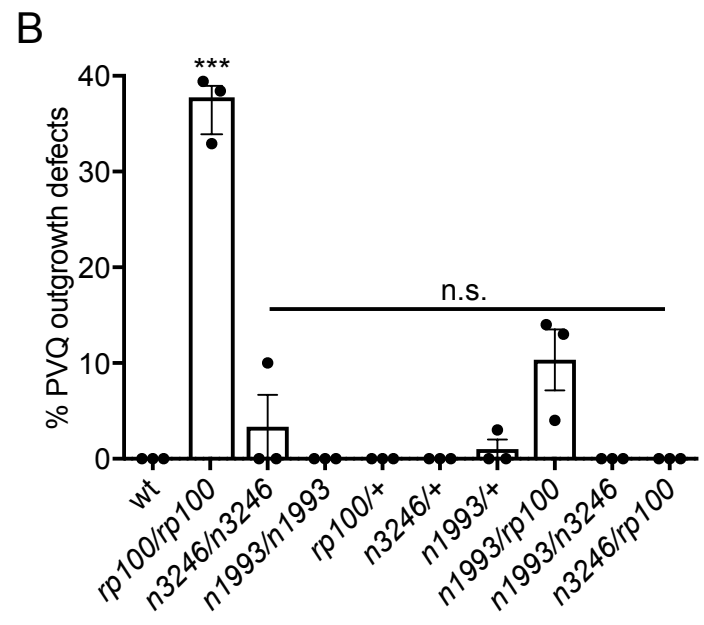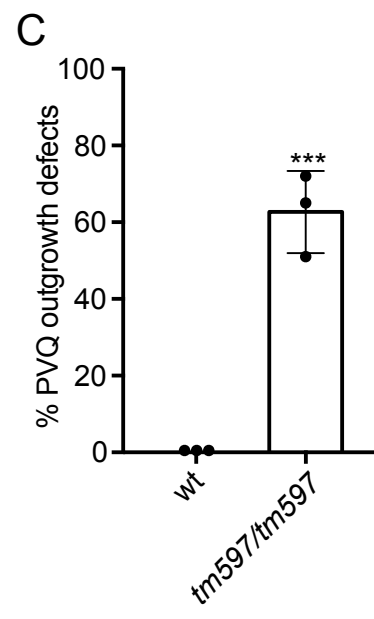

Supplement: S2 Fig — genetic analysis of ced-10 alleles. (A) Quantification of total PVQ defects (outgrowth and guidance) in wild type, ced-10 mutant homozygotes, heterozygotes and transheterozygotes. Data are expressed as mean ±SD and statistical significance was assessed using one-way ANOVA, with Tukey’s multiple comparison test. ****<0.0001, n.s. not significantly different to wild type. n = 90 per strain, each dot represents independent scoring replicates. (B) Quantification of PVQ outgrowth defects in wild type, ced-10 mutant homozygotes, heterozygotes and transheterozygotes. Data are expressed as mean ±SD and statistical significance was assessed using one-way ANOVA, with Tukey’s multiple comparison test. ****<0.0001, n.s. not significantly different to wild type. n = 90 per strain, each dot represents independent scoring replicates. (C) Quantification of PVQ outgrowth defects in wild type L1 larvae and ced-10(tm597) L1 escapers. Data are expressed as mean ±SD and statistical significance was assessed using t test. n = 9 per strain, each dot represents independent scoring replicates. (PDF) [file pgen.1007670.s002.pdf]

A

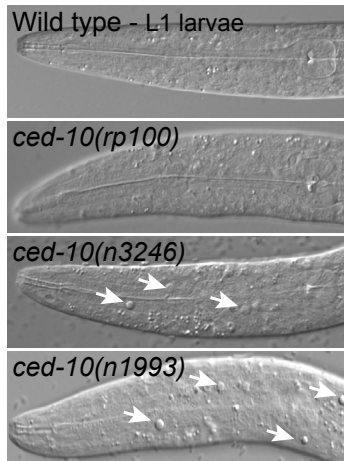

B

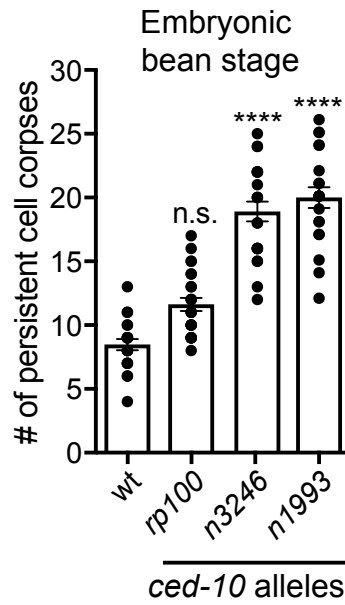

C

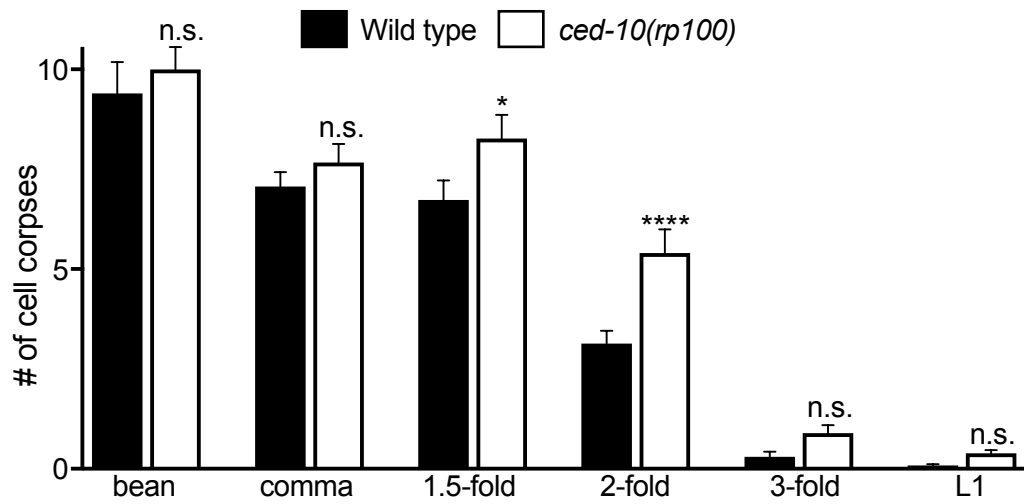

D

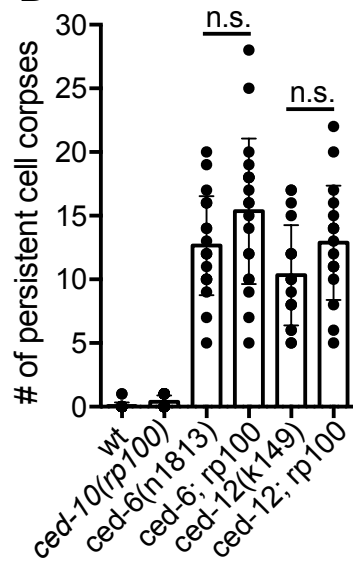

E

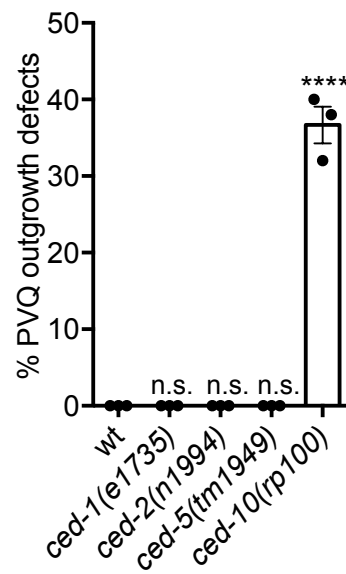

F

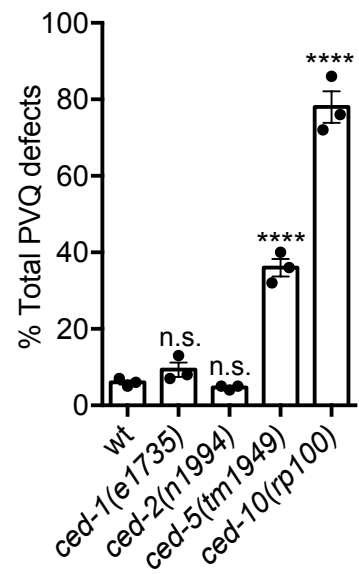

Supplement: S3 Fig — apoptotic pathway function. (A) Images of the heads of freshly hatched L1 animals. Persistent apoptotic cell corpses are marked with white arrows. (B) Quantification of apoptotic corpse engulfment in ced-10 mutant animals. The number of persistent cell corpses was counted at the bean stage of embryogenesis. Data are expressed as mean ±SD and statistical significance was assessed using one-way ANOVA, with Tukey’s multiple comparison test. ****<0.0001, n.s., not statistically significant from wild type. n = 21, from three independent scorings. (C) Quantification of apoptotic corpse engulfment in wild type and ced-10(rp100) mutant animals. Data are expressed as mean ±SD and statistical significance was assessed using one-way ANOVA, with Tukey’s multiple comparison test. ****<0.0001, *<0.01, n.s., not statistically significant from wild type. n = 15, from three independent scorings. (D) Quantification of persistent cell corpses present in the head of freshly hatched ced-10(rp100) L1 larvae combined with mutations in CED-6/GULP and CED-12/ELMO. Data are expressed as mean ±SD and statistical significance was assessed using one-way ANOVA, with Tukey’s multiple comparison test. n.s. not statistically significant comparing ced-6 and ced-12 single mutants combined with ced-10(rp100). n = 17, from three independent scorings. (E) Quantification of PVQ outgrowth defects in wild type and mutants of apoptotic pathway genes. Data are expressed as mean ±SD and statistical significance was assessed using one-way ANOVA, with Tukey’s multiple comparison test. ****<0.0001, n.s. not significantly different to wild type. n = 75 per strain, each dot represents independent scoring replicates. (F) Quantification of total PVQ defects (outgrowth and guidance) in wild type and mutations in apoptotic pathway genes. Data are expressed as mean ±SD and statistical significance was assessed using one-way ANOVA, with Tukey’s multiple comparison test. ****<0.0001, n.s. not significantly different to w [file pgen.1007670.s003.pdf]

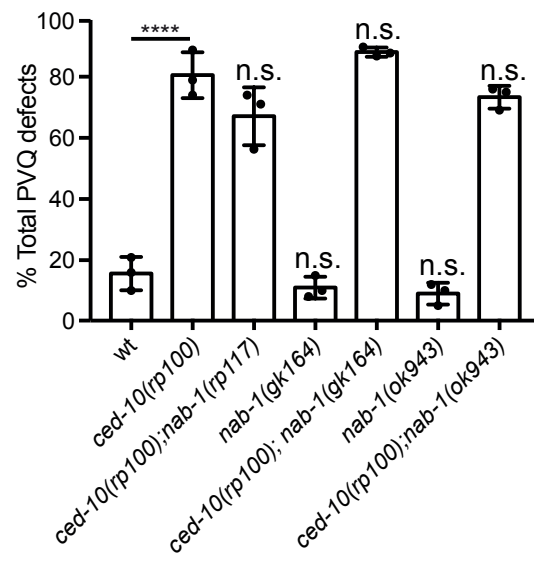

Supplement: S4 Fig — genetic analysis of NAB-1 function in PVQ development. Quantification of total PVQ defects (outgrowth and guidance) in wild type, ced-10(rp100) and compound mutants between ced-10(rp100) and nab-1 alleles (rp117, gk164 and ok943). Data are expressed as mean ±SD and statistical significance was assessed using one-way ANOVA, with Tukey’s multiple comparison test. ****<0.0001, comparing wild type and rp100 animals, n.s. not statistically significant when compared to either wild type (for single mutants) or ced-10(rp100) for compound mutants. n = 90 per strain, each dot represents independent scoring replicates. (PDF) [file pgen.1007670.s004.pdf]
