# Supplementary material for: Distinct CED-10/Rac1 domains confer context-specific functions in development
Source: PLoS Genet. 2018 Sep 28;14(9):e1007670. doi: 10.1371/journal.pgen.1007670 (PMC6179291; doi:10.1371/journal.pgen.1007670)
Supplement: S1 Table — (PDF) [file pgen.1007670.s005.pdf]

Table S1. Genetic Analysis of Candidate CED-10/Rac1 Regulatory and Effector Molecules

	Single mutant outgrowth defect (%)	Double *ced-10(rp100)* mutant outgrowth phenotype (%)	Single mutant outgrowth/guidance phenotype (%)	Double *ced-10(rp100)* mutant outgrowth/ guidance phenotype (%)	Mammalian Homolog
ced-10(rp100)	37	-	84	-	Rac1 GTPase
Effectors/Interactors					
max-2(ok1904)	1	35 n.s.	61	91 n.s.	*pak-1* paralog
mig-10(ct41)	2	27 n.s.	75	88 n.s.	Lamellipodin
pak-1(ok448)	0	13 ***	4	90 n.s..	p21 MAPK
pak-1(tm403)	0	5 ****	5	78 n.s.	p21 MAPK
pak-2(ok332)	0	31 n.s.	7	87 n.s.	p21 MAPK
rin-1(gk431)	0	38 n.s.	6	93 n.s.	RIN (Ras interactor)
unc-53(n152)	88	91 n.s.	100	100 n.s.	NAV2
unc-53(e2432)	84	86 n.s.	98	100 n.s.	NAV2
unc-115(ky275)	0	40 n.s.	19	95**	abLIM
Regulators					
tiam-1(ok772)	0	33 n.s.	12	75 n.s.	TIAM1
unc-73(e936)	55	56 n.s.	100	100 n.s.	Trio-like GEF
srgp-1(gk3017)	0	14 ****	10	83 n.s.	srGAP
syd-1(ju82)	0	12 ****	0	83 n.s.	GAP-like
syd-1(tm6234)	0	18 ****	0	76 n.s.	GAP-like
nab-1(rp117)	n.d.	10 ****	n.d.	86 n.s.	Neurabin
nab-1(gk164)	0	21 ***	0	78 n.s.	Neurabin
nab-1(ok943)	0	5 ****	0	76 n.s.	Neurabin
Rac GTPases					
mig-2(mu28)	4	73 ****	66	100**	RhoG
rac-2(ok326)	0	27 n.s.	9	83 n.s.	Rac1 GTPase
Apoptotic Pathway Genes					
ced-1(e1735)	0	n.d.	9	n.d.	RhoG
ced-2(n1994)	0	n.d.	4	n.d.	Rac1 GTPase
ced-5(tm1949)	0	n.d.	35	n.d.	RhoG
