# Supplementary material for: Distinct CED-10/Rac1 domains confer context-specific functions in development
Source: PLoS Genet. 2018 Sep 28;14(9):e1007670. doi: 10.1371/journal.pgen.1007670 (PMC6179291; doi:10.1371/journal.pgen.1007670)
Supplement: S2 Table — (PDF) [file pgen.1007670.s006.pdf]

Table S2. Neuroanatomical analysis of *ced-10* mutant function

	% defective animals			
**Neurons examined (marker used)**	**wild-type**	***ced-10 (rp100)***	***ced-10 (n3246)***	***ced-10 (n1993)***
Interneurons				
PVQs (*oyIs14*)				
Guidance[a]/Outgrowth[b]	5%	84%****	81%****	44%****
Guidance[a]	5%	65%****	81%****	44%****
Outgrowth[b]	0%	37%***	3% n.s	0% n.s
PVPs (*hdIs26*)				
Guidance[a]	6%	97%****	66%****	51%****
AVG (*otIs182*)				
Branching[c]	0%	0%	24%****	0%
Outgrowth[d]	9%	100%****	44%****	56%****
Motor Neurons				
HSNs (*rpEx6*)				
Guidance[a]	5%	85%****	72%****	52%****
Migration[f]	3%	40%****	40%****	53%****
DDs (*oxIs12*)				
L/R choice[g]	0%	27%*	27%*	7% n.s
Commissural guidance[h]	0%	93%****	60%****	0% n.s
VDs/DDs (*oxIs12*)				
L/R choice[g]	13%	80%****	87%****	77%****
Commissural guidance[h]	3%	93%****	61%****	3% n.s
Mechanosensory neurons				
PDEs (*lqIs2*)				
Guidance[i]	7%	4% n.s	18%**	8% n.s
PLMs (*zdIs5*)				
mispositoned cell body[j]	6%	14% n.s	47%***	4% n.s
Outgrowth[k]	0%	25%****	79%****	24%****
Guidance[l]	5%	7% n.s	54%**	12% n.s
ALMs (*zdIs5*)				
Synapse formation[m]	3%	35%****	89%****	11% n.s
Outgrowth[n]	0%	3% n.s	24%***	5% n.s
PVM (*zdIs5*)				
mispositioned cell body[o]	1%	14%*	66%****	17%**
Guidance[p]	3%	6% n.s	24%**	18%**
AVM (*zdIs5*)				
Outgrowth[n]	0%	16%****	65%****	4%*
Sensory Neurons (*rpIs8*)				
AQR				
Migration[q]	7%	9% n.s	19%*	7% n.s

[a]Axons inappropriately cross the midline or fail to defasciculate.
[b]Axons not reaching the nerve ring of the animal and stopping in either the right or left ventral fascicle. The majority of outgrowth defects occur with a guidance defect (78%).
[c]Axon with ectopic branches after the pre-anal ganglion.
[d]Axon fail to extend beyond the pre-anal ganglion.
[f]Cell body fails migrate to the target position before the vulva.
[g]Axons extend commissures on the inappropriate side of the animal.
[h]Axons take winding path before reaching the dorsal nerve cord.
[i]Axons inappropriately cross the midline and/or extend anteriorly before reaching the VNC.
[j]Cell body is two or more cell body lengths anterior or posterior of correct position.
[k]Axons failing to reach their target between the vulva and AVM cell body.
[l]Axons extends inappropriately into the VNC or the DNC.
[m]Axons do not form synapse into the nerve ring.
[n]Axons fail to extend beyond the nerve ring.
[o]Cell body mispositioned on PLML axon.
[p]Axon extends posteriorly before reaching the VNC.
[q]Cell body does not reach target position, just posterior to the posterior pharyngeal bulb.
