# Supplementary material for: Distinct CED-10/Rac1 domains confer context-specific functions in development
Source: PLoS Genet. 2018 Sep 28;14(9):e1007670. doi: 10.1371/journal.pgen.1007670 (PMC6179291; doi:10.1371/journal.pgen.1007670)
Supplement: S3 Table — (PDF) [file pgen.1007670.s007.pdf]

Table S3. List of strains used in this study

Strain	Genotype	Transgene	Reference
N2			[1]
CX5334	*oyIs14 V*	*sra-6*^{prom}*::GFP; lin-15(+)*	Sengupta lab
SK4005	*zdIs5 I*	*mec-4*^{prom}*::GFP; lin-15(+)*	[2]
LE309	*lqIs2 X*	*osm-6*^{prom}*::GFP; lin-15(+)*	[3]
RJP280	*rpIs8*	*gcy-33*^{prom}*::GFP*	This study
OH4887	*otIs182*	*inx-18*^{prom}*::GFP*	Hobert lab
EG1306	*oxIs12 X*	*unc-47*^{prom}*::GFP; lin-15(+)*	[4]
VH648	*hdIs26 III*	*odr-2*^{prom}*::cfp; sra-6*^{prom}*::DsRed2*	[5]
RJP18	*rpEx6*	*tph-1*^{prom}*::GFP*	This study
RJP3544	*rpEx1640*	*sra-6*^{prom}*::GFP*	This study

ced-10 alleles - survey of neuronal development (all this study)

RJP3004	*ced-10(rp100) IV; oyIs14 V*
RJP3023	*ced-10(n1993) IV; oyIs14 V*
RJP3134	*ced-10(n3246) IV; oyIs14 V*
RJP3838	*ced-10(tm597) / dpy-13(e184) IV; oyIs14 V*
RJP3357	*ced-10(knu268) IV; oyIs14 V*
RJP3135	*ced-10(rp100) IV; rpEx6*
RJP3142	*ced-10(n1993) IV; rpEx6*
RJP3140	*ced-10(n3246) IV; rpEx6*
RJP3348	*ced-10(rp100) IV; otIs182*
RJP3264	*ced-10(rn1993) IV; otIs182*
RJP3265	*ced-10(n3246) IV; otIs182*
RJP3154	*ced-10(rp100) IV; lqIs2 X*
RJP3147	*ced-10(rn1993) IV; lqIs2 X*
RJP3141	*ced-10(n3246) IV; lqIs2 X*
RJP3168	*ced-10(rp100) IV; rpIs8*
RJP3143	*ced-10(n1993) IV; rpIs8*
RJP3167	*ced-10(n3246) IV; rpIs8*
RJP3138	*ced-10(rp100) IV; zdIs5 I*
RJP3185	*ced-10(n1993) IV; zdIs5 I*
RJP3139	*ced-10(n3246) IV; zdIs5 I*
RJP3144	*ced-10(rp100) IV; oxIs12 X*
RJP3148	*ced-10(n1993) IV; oxIs12 X*
RJP3137	*ced-10(n3246) IV; oxIs12 X*

RJP3349	*ced-10(rp100) IV; hdIs26 III*	
RJP3322	*ced-10(n1993) IV; hdIs26 III*	
RJP3323	*ced-10(n3246) IV; hdIs26 III*	
**Cell corpse engulfment mutants**		
RJP1941	*ced-10(rp100) IV*	This study
MT5013	*ced-10(n1993) IV*	[6]
MT9958	*ced-10(n3246) IV*	[7]
NF87	*ced-12(k149) I*	[8]
RJP3324	*ced-12(k149) I; ced-10(rp100) IV*	This study
RJP3156	*lon-1(e185) ced-6(n1813) III*	This study
RJP3155	*lon-1(e185) ced-6(n1813) III; ced-10(rp100) IV*	This study
**Candidate PVQ regulators (all this study)**		
RJP3456	*max-2(ok1904) II; oyIs14 V*	
RJP3459	*max-2(ok1904) II; max-2(ok1904) oyIs14 V*	
RJP3467	*mig-10(ct41) III; oyIs14 V*	
RJP3471	*mig-10(ct41) III; ced-10(rp100) oyIs14 V*	
RJP3354	*pak-1(ok448) X; oyIs14 V*	
RJP3355	*ced-10(rp100) IV; pak-1(ok448) X; oyIs14 V*	
RJP3503	*pak-1(tm403) X; oyIs14 V*	
RJP3481	*ced-10(rp100) IV; pak-1(tm403) X; oyIs14 V*	
RJP3461	*pak-2(ok332) X; hdIs26 III*	
RJP3480	*ced-10(rp100) IV; pak-2(ok332) X; hdIs26 III*	
RJP3486	*rin-1(gk431) V; hdIs26 III*	
RJP3191	*ced-10(rp100) IV; rin-1(gk431) V; hdIs26 III*	
RJP3509	*ced-10(rp100) IV; slt-1(eh15) X; oyIs14 V*	
RJP3347	*tiam-1(ok772) I; oyIs14 V*	
RJP3346	*tiam-1(ok772) I; ced-10(rp100) IV; oyIs14 V*	
RJP3307	*unc-53(e2432) II; oyIs14 V*	
RJP3345	*unc-53(e2432) II; ced-10(rp100) IV; oyIs14 V*	
RJP3305	*unc-53(n152) II; oyIs14 V*	
RJP3344	*unc-53(n152) II; ced-10(rp100) IV; oyIs14 V*	
RJP3360	*unc-73(e936) I; oyIs14 V*	
RJP3504	*unc-73(e936) I; ced-10(rp100) IV; oyIs14 V*	
RJP3474	*unc-115(ky275) X; oyIs14 V*	
RJP3475	*ced-10(rp100) IV; unc-115(ky275) X; oyIs14 V*	

RJP3364	*mig-2(mu28) X; oyIs14 V*
RJP3306	*rac-2(ok326) X; oyIs14 V*
RJP3366	*ced-10(rp100) IV; mig-2(mu28) X; oyIs14 V*
RJP3343	*ced-10(rp100) IV; rac-2(ok326) X; oyIs14 V*
RJP3473	*mig-2(mu28) rac-2(ok326) X; oyIs14 V*
RJP3490	*ced-1(e1735) I; oyIs14 V*
RJP3494	*ced-2(n1994) IV; oyIs14 V*
RJP3538	*ced-5(tm1949) IV; oyIs14 V*
RJP3302	*ced-10(nu978) IV; oyIs14 V*
RJP3724	*nab-1(rp117) I; ced-10(rp100) IV; oyIs14 V*
RJP3728	*nab-1(gk164) I; oyIs14 V*
RJP3729	*nab-1(gk164) I; ced-10(rp100) IV; oyIs14 V*
RJP3730	*nab-1(ok943) I; oyIs14 V*
RJP3731	*nab-1(ok943) I; ced-10(rp100) IV; oyIs14 V*
RJP3732	*srgp-1(gk3017) IV; oyIs14 V*
RJP3733	*ced-10(rp100) srgp-1(gk3017) IV; oyIs14 V*
RJP3827	*syd-1(tm6234) II; ced-10(rp100) IV; oyIs14 V*
RJP3828	*syd-1(tm6234) II; oyIs14 V*
RJP3881	*syd-1(ju82) II; ced-10(rp100) IV; oyIs14 V*
RJP3880	*syd-1(ju82) II; oyIs14 V*
RJP3896	*nab-1(ok943) I; syd-1(ju82) II; ced-10(rp100) IV; oyIs14 V*
RJP3898	*syd-1(ju82) II; ced-10(rp100) IV; oyIs14 V; mig-2(mu28) X*
RJP3836	*nab-1(ok943) I; ced-10(rp100) IV; oyIs14 V; mig-2(mu28) X*

Transgenic Rescue lines (all this study)

RJP3105	*ced-10(rp100) IV; oyIs14 V; rpEx1528*	*WRM0639dH09-ced-10(+)*<br>*myo-2^{prom}::mCherry* Line 1
RJP3108	*ced-10(rp100) IV; oyIs14 V; rpEx1530*	*WRM0639dH09-ced-10(+)*<br>*myo-2^{prom}::mCherry* Line 2
RJP3109	*ced-10(rp100) IV; oyIs14 V; rpEx1531*	*WRM0639dH09-ced-10(+)*<br>*myo-2^{prom}::mCherry* Line 3
RJP3308	*ced-10(rp100) IV; oyIs14 V; rpEx1582*	*sra-6^{prom}::ced-10cDNAb*<br>*myo-2^{prom}::mCherry* Line 1
RJP3309	*ced-10(rp100) IV; oyIs14 V; rpEx1583*	*sra-6^{prom}::ced-10cDNAb*<br>*myo-2^{prom}::mCherry* Line 2
RJP3310	*ced-10(rp100) IV; oyIs14 V; rpEx1584*	*sra-6^{prom}::ced-10cDNAb*<br>*myo-2^{prom}::mCherry* Line 3
RJP3250	*ced-10(rp100) IV; oyIs14 V; rpEx1565*	*rgef-1^{prom}::ced-10cDNAb*<br>*myo-2^{prom}::mCherry* Line 1
RJP3251	*ced-10(rp100) IV; oyIs14 V; rpEx1566*	*rgef-1^{prom}::ced-10cDNAb*<br>*myo-2^{prom}::mCherry* Line 2
RJP3252	*ced-10(rp100) IV; oyIs14 V; rpEx1567*	*rgef-1^{prom}::ced-10cDNAb*<br>*myo-2^{prom}::mCherry* Line 3
RJP3507	*ced-10(rp100) IV; oyIs14 V; rpEx1625*	*rgef-1^{prom}::ced-10cDNAb*<br>*myo-2^{prom}::mCherry*<br>*sra-6^{prom}::mCherry*
RJP3897	*nab-1(ok943) I; ced-10(rp100) IV; oyIs14 V; rpEx1666*	*npr-11^{prom}::nab-1cDNA*<br>*myo-2^{prom}::mCherry*

RJP3900	*nab-1(ok943) I; ced-10(rp100) IV; oyIs14 V; rpEx1667*	*npr-11^{prom}::nab-1cDNA myo-2^{prom}::mCherry*

1. Brenner, S. The genetics of Caenorhabditis elegans. *Genetics* **77**, 71-94 (1974).
2. Clark, S.G. & Chiu, C. C. elegans ZAG-1, a Zn-finger-homeodomain protein, regulates axonal development and neuronal differentiation. *Development* **130**, 3781-3794 (2003).
3. Struckhoff, E.C. & Lundquist, E.A. The actin-binding protein UNC-115 is an effector of Rac signaling during axon pathfinding in C. elegans. *Development* **130**, 693-704 (2003).
4. McIntire, S.L., Reimer, R.J., Schuske, K., Edwards, R.H. & Jorgensen, E.M. Identification and characterization of the vesicular GABA transporter. *Nature* **389**, 870-876 (1997).
5. Hutter, H. Extracellular cues and pioneers act together to guide axons in the ventral cord of C. elegans. *Development* **130**, 5307-5318 (2003).
6. Ellis, R.E., Jacobson, D.M. & Horvitz, H.R. Genes required for the engulfment of cell corpses during programmed cell death in Caenorhabditis elegans. *Genetics* **129**, 79-94 (1991).
7. Reddien, P.W. & Horvitz, H.R. CED-2/CrkII and CED-10/Rac control phagocytosis and cell migration in Caenorhabditis elegans. *Nat Cell Biol* **2**, 131-136 (2000).
8. Chung, S., Gumienny, T.L., Hengartner, M.O. & Driscoll, M. A common set of engulfment genes mediates removal of both apoptotic and necrotic cell corpses in C. elegans. *Nat Cell Biol* **2**, 931-937 (2000).
