# Supplementary material for: Distinct CED-10/Rac1 domains confer context-specific functions in development
Source: PLoS Genet. 2018 Sep 28;14(9):e1007670. doi: 10.1371/journal.pgen.1007670 (PMC6179291; doi:10.1371/journal.pgen.1007670)
Supplement: S4 Table — (PDF) [file pgen.1007670.s008.pdf]

Table S4. List of mutant alleles used in this study

Gene	Allele	Nature of allele	Reference
ced-10	*rp100*	Gly to Glu amino acid substitution residue 30	This study
ced-10	*knu268*	Pro to Ser amino acid substitution residue 29	This study
ced-10	*n3246*	Gly to Arg amino acid substitution residue 60	[1]
ced-10	*n1993*	Val to Gly amino acid substitution residue 190	[1]
ced-1	*e1735*	Gln to opal stop codon at position 375, cell corpse phenotype comparable to the null *e1754*	[2]
ced-2	*n1994*	Arg to opal stop codon at residue 102	[1]
ced-5	*tm1949*	presumptive null	[3]
ced-6	*n1813*	deletion of one nucleotide in codon 360 leading to frameshift and loss of 132 amino acids	[4]
ced-12	*k149*	Arg to opal stop codon at residue 38	[5]
max-2	*ok1904*	2200 dp deletion removes the third, fourth and most of the fifth last exon in all isoforms	
mig-2	*mu28*	Trp to opal stop codon at residue 60, presumptive null	[6]
mig-10	*ct41*	Opal stop codon in exon 3 presumptive null	[7]
nab-1	*rp117*	Premature stop codon at residue 652 out of 721	This study
nab-1	*gk164*	492 bp in-frame deletion, including part of actin binding domain	[8]
nab-1	*ok943*	1032 bp out of frame deletion disrupting the 3 last exons	[8]
pak-1	*ok448*	1445 bp deletion removing kinase domain and CRIB domain	[9]
pak-1	*tm403*	1474 bp deletion + 15bp addition removing kinase domain and CRIB domain	[9]
pak-2	*ok332*	Removes start codon	[10]
rac-2	*ok326*	Exon 2-4 removed, presumptive null	[11]
rin-1	*gk431*	Removes part of the third last exon in all isoforms. Considered strong loss of function.	[12]
srgp-1	*gk3017*	1203 bp deletion including RhoGAP domain	
syd-1	*tm6234*	1264bp out of frame deletion	
syd-1	*ju82*	Premature stop codon, putative null	[13]
tiam-1	*ok772*	838 bp deletion, strong loss of function removes some of the DH and PH domain	[14]
unc-53	*e2432*	375 bp deletion, missing a part of exon 12	[15]
unc-53	*n152*	374 bp deletion, missing a part of exon 18	[15]
unc-73	*e936*	splice site mutation causing severe reduction of wild type protein	[16]
unc-115	*ky275*	Trp to amber stop codon at residue 489, presumptive null	[17]

1. Reddien, P.W. & Horvitz, H.R. CED-2/CrkII and CED-10/Rac control phagocytosis and cell migration in Caenorhabditis elegans. *Nat Cell Biol* **2**, 131-136 (2000).
2. Zhou, Z., Hartwieg, E. & Horvitz, H.R. CED-1 Is a Transmembrane Receptor that Mediates Cell Corpse Engulfment in C. elegans. *Cell* **104**, 43-56 (2001).

3. Neukomm, L.J., *et al.* The phosphoinositide phosphatase MTM-1 regulates apoptotic cell corpse clearance through CED-5-CED-12 in C. elegans. *Development* **138**, 2003-2014 (2011).
4. Liu, Q.A. & Hengartner, M.O. Candidate adaptor protein CED-6 promotes the engulfment of apoptotic cells in C. elegans. *Cell* **93**, 961-972 (1998).
5. Gumienny, T.L., *et al.* CED-12/ELMO, a Novel Member of the CrkII/Dock180/Rac Pathway, Is Required for Phagocytosis and Cell Migration. *Cell* **107**, 27-41 (2001).
6. Zipkin, I.D., Kindt, R.M. & Kenyon, C.J. Role of a New Rho Family Member in Cell Migration and Axon Guidance in C. elegans. *Cell* **90**, 883-894 (1997).
7. Manser, J., Roonprapunt, C. & Margolis, B. C. elegansCell Migration Genemig-10Shares Similarities with a Family of SH2 Domain Proteins and Acts Cell Nonautonomously in Excretory Canal Development. *Developmental Biology* **184**, 150-164 (1997).
8. Hung, W., Hwang, C., Po, M.D. & Zhen, M. Neuronal polarity is regulated by a direct interaction between a scaffolding protein, Neurabin, and a presynaptic SAD-1 kinase in Caenorhabditis elegans. *Development* **134**, 237-249 (2007).
9. Zhang, H., *et al.* A tension-induced mechanotransduction pathway promotes epithelial morphogenesis. *Nature* **471**, 99-103 (2011).
10. Lucanic, M., Kiley, M., Ashcroft, N., L'Etoile, N. & Cheng, H.-J. The Caenorhabditis elegans P21-activated kinases are differentially required for UNC-6/netrin-mediated commissural motor axon guidance. *Development* **133**, 4549-4559 (2006).
11. Modzelewska, K., *et al.* An Activating Mutation in sos-1 Identifies Its Dbl Domain as a Critical Inhibitor of the Epidermal Growth Factor Receptor Pathway during Caenorhabditis elegans Vulval Development. *Molecular and Cellular Biology* **27**, 3695-3707 (2007).
12. Doi, M., Minematsu, H., Kubota, Y., Nishiwaki, K. & Miyamoto, M. The novel Rac effector RIN-1 regulates neuronal cell migration and axon pathfinding in C. elegans. *Development* **140**, 3435-3444 (2013).
13. Hallam, S.J., Goncharov, A., McEwen, J., Baran, R. & Jin, Y. SYD-1, a presynaptic protein with PDZ, C2 and rhoGAP-like domains, specifies axon identity in C. elegans. *Nat Neurosci* **5**, 1137-1146 (2002).
14. Demarco, R.S., Struckhoff, E.C. & Lundquist, E.A. The Rac GTP Exchange Factor TIAM-1 Acts with CDC-42 and the Guidance Receptor UNC-40/DCC in Neuronal Protrusion and Axon Guidance. *PLoS Genet* **8**, e1002665 (2012).
15. Stringham, E., Pujol, N., Vandekerckhove, J. & Bogaert, T. unc-53 controls longitudinal migration in C. elegans. *Development* **129**, 3367-3379 (2002).
16. Steven, R., *et al.* UNC-73 Activates the Rac GTPase and Is Required for Cell and Growth Cone Migrations in *C. elegans*. *Cell* **92**, 785-795 (1998).
17. Lundquist, E.A., Herman, R.K., Shaw, J.E. & Bargmann, C.I. UNC-115, a conserved protein with predicted LIM and actin-binding domains, mediates axon guidance in C. elegans. *Neuron* **21**, 385-392 (1998).
