# Supplementary material for: Distinct CED-10/Rac1 domains confer context-specific functions in development
Source: PLoS Genet. 2018 Sep 28;14(9):e1007670. doi: 10.1371/journal.pgen.1007670 (PMC6179291; doi:10.1371/journal.pgen.1007670)
Supplement: S5 Table — (PDF) [file pgen.1007670.s009.pdf]

Table S5. List of genotyping primers used in this study

Gene	Primer	Sequence	Band size
ced-10(rp100)	JDE33	tatcctacaccacaaacgcatttccaga	Four primer PCR
	JDE34	aaaaatgactcaccgtcggaatatattatc	386bp (wt)
	JDE41	gctcgcttttctctcgaaattcgatgac	319bp (mutant)
	JDE42	ttatcgcattttgaaaagttttttgtctag	
ced-10(P29S)	CEH4283	cgtcttgatgcccgttgtgatt	665bp (Sanger sequence)
	CEH4284	tcactatttttcccagtttttggcct	
ced-12(k149)	SNO237	gtcgaaaaagtttcgtttttttcaag	881bp (Sanger sequence)
	SNO238	cgattaaatattctggaaactatag	
max-2(ok1904)	SNO277	cgcgttgcttgatctctggc	833bp (wt)
	SNO278	ttgattgaggtacatttaaagccg	no PCR product (mutant)
mig-2(mu28)	SNO273	cgttcaccattagtgttatttcc	775bp (Sanger sequence)
	SNO274	ggaaacaaccatttctgagcc	
pak-1(ok448)	JLW11	ggtttggcgcatcggaggaa	510bp (wt)
	JLW12	tgagaactaccgaaagctggg	no PCR product (mutant)
pak-1(tm403)	JDE56	cgtatgatgagaaaccaccag	1025bp (wt)
	JDE57	gtgagtctgtgagaagacgtg	no PCR product (mutant)
pak-2(ok332)	SNO281	gcataaaaggcagtgtggtgtc	621bp (wt)
	SNO282	gtgtggcttctcattttggagg	no PCR product (mutant)
rac-2(ok326)	SNO252	ctgaaagtggaaaattttgaccg	733bp (wt)
	SNO253	cgtctgtttgtggaaatgacag	no PCR product (mutant)
rin-1(gk431)	SNO295	gagaatgtagacaaaccagcg	746bp (wt)
	SNO296	gttcttcataaacttttgctccg	no PCR product (mutant)
tiam-1(ok772)	SNO250	tctgtgtatgagacaagtccga	628bp (wt)
	SNO251	atgaatactgcacacactcgc	no PCR product (mutant)
nab-1(gk164)	JDE157	atctcaatgtccgccgagtc	522bp (wt)
	JDE158	aaatactttggcccgcgaac	no PCR product (mutant)
nab-1(ok943)	JDE155	gatatcgaagagccgccacc	~800bp (wt)
	JDE156	acaaacatcgtccaccgtcc	no PCR product (mutant)
syd-1(ju82)	JDE211	attcaaagactcaccgggaggcggatc	Four primer PCR
	JDE212	ctcacgcagtcgcatgaatgccactc	341bp (wt)
	JDE213	cgagctcagagttcgagcacttagtccctg	250bp (mutant
	JDE214	gattgcaggactagccacgcgaaaaagg	
syd-1(tm6234)	JDE185	ttaagcccacgtccttcgac	2187bp (wt)
	JDE188	aatcgtcctttcccgagag	923bp (mutant)
